# Development of an Aerial Manipulation System Using Onboard Cameras and a Multi-Fingered Robotic Hand with Proximity Sensors

**DOI:** 10.3390/s25020470

**Published:** 2025-01-15

**Authors:** Ryuki Sato, Etienne Marco Badard, Chaves Silva Romulo, Tadashi Wada, Aiguo Ming

**Affiliations:** Department of Mechanical and Intelligent Systems Engineering, The University of Electro-Communications, Tokyo 1828585, Japan

**Keywords:** aerial manipulation, unmanned aerial vehicle flight control, tracking camera, depth camera, robotic hand, proximity sensors

## Abstract

Recently, aerial manipulations are becoming more and more important for the practical applications of unmanned aerial vehicles (UAV) to choose, transport, and place objects in global space. In this paper, an aerial manipulation system consisting of a UAV, two onboard cameras, and a multi-fingered robotic hand with proximity sensors is developed. To achieve self-contained autonomous navigation to a targeted object, onboard tracking and depth cameras are used to detect the targeted object and to control the UAV to reach the target object, even in a Global Positioning System-denied environment. The robotic hand can perform proximity sensor-based grasping stably for an object that is within a position error tolerance (a circle with a radius of 50 mm) from the center of the hand. Therefore, to successfully grasp the object, a requirement for the position error of the hand (=UAV) during hovering after reaching the targeted object should be less than the tolerance. To meet this requirement, an object detection algorithm to support accurate target localization by combining information from both cameras was developed. In addition, camera mount orientation and UAV attitude sampling rate were determined by experiments, and it is confirmed that these implementations improved the UAV position error to within the grasping tolerance of the robot hand. Finally, the experiments on aerial manipulations using the developed system demonstrated the successful grasping of the targeted object.

## 1. Introduction

Aerial manipulation, combining unmanned aerial vehicle (UAV) technology and robotic manipulators engaging in physical interactions while in flight, holds the potential to revolutionize logistics, transportation, facility management, and various other industries. A wide range of research areas have been developed for aerial manipulation, including object recognition, self-localization, map generation and navigation, and manipulator and mechanism design [1,2].

One of the significant challenges in aerial manipulation is that the distinctive UAV system, which has a floating reference frame, is subject to external disturbances such as natural wind and ground effects. Oscillations of the UAV body induce position errors between the object to be grasped and the hand leading to grasp failure or damage to the grasped object due to unexpected collisions with the fingers. In recent years, there has been an increase in aerial grasping hands that use flexible fingers to mitigate the impact of collisions between the object and the fingers [3,4,5]. In theory, positioning errors of the UAV within these hand-opening widths are tolerated; however, it is difficult to avoid unexpected finger collisions during the grasping motion if it has a slight position error which could lead to displacement or damage to the object.

Our research group has developed a multi-fingered robotic hand with proximity sensors to achieve aerial manipulation with stable grasping by addressing such position errors [6]. The optical proximity sensors attached to the palm and the fingertips of the robotic hand detects the proximal object surface without contact. Aerial manipulation by a UAV hovering near a targeted object was successfully performed using flight positioning and grasping controls based on these proximity sensors. However, because the developed system required the UAV’s self-location information for flight control, a ground-based motion capture system was employed. In addition, object recognition was not considered. Therefore, the next challenges involve object detection, encompassing object identification and localization and flight positioning to guide the UAV from a wide area into the grasping range of the robotic hand without using an external system.

The Global Positioning System (GPS), a representative positioning technology for UAV flight control, is used in many studies [7,8] and also in drone products by DJI (Shenzhen, China) and Parrot (Paris, France), two of the leading manufacturers in the drone market. In GPS-based flight positioning, Real-Time Kinematic GPS (RTK-GPS), which incorporates correctional position information from ground base stations to improve positioning accuracy, is often utilized and enables positioning flights with an accuracy of approximately 1–5 cm [9,10]. However, it cannot be used indoors, in caves, or in forests, where GPS signals are problematic to reach. For the localization of UAVs for indoor flight, a vision-based motion capture system is often employed, which captures the target UAV using cameras installed in the environment [11,12]. Motion capture typically enables highly accurate location data; however, there are issues around limiting the UAV’s flying area within the detection range and its prohibitive introduction cost.

Research is being actively conducted into technologies for self-localization and environmental mapping using onboard sensors, such as Light Detection And Ranging (LiDAR) sensors or vision sensors, for unmanned vehicles in GPS-denied environments [13,14,15]. LiDAR measures the distance to an object and determines its shape by calculating the time difference between emitting a laser beam and receiving its reflection. It is often used in conjunction with a pre-designed map to estimate self-location. In autonomous aerial manipulation, cameras are more versatile than being used solely for positioning; they can also be used for object detection and identification. Several studies [16,17,18,19] have utilized single- or multi-vision sensor(s) for Visual–Inertial Odometry (VIO) and Simultaneous Localization and Mapping (SLAM) in drones. VIO is a method for estimating a moving path by fusing data from camera(s) and an Inertial Measurement Unit (IMU), which typically includes an accelerometer and a gyroscope. These research efforts allowed for drones to create detailed maps and achieve precise localization or object avoidance in complex environments; however, these studies primarily focused on navigation and mapping. Li et al. [20] evaluated the accuracy of the drone flight based on VIO by fusing RGB-D visual odometry using an onboard Kinect camera and motion estimation from IMU and reported that the maximum indoor flight position deviation was approximately 8 cm. Gerwen et al. [14] surveyed studies on positioning using sensors fusionally, such as visual, inertial, and ultrasonic sensors, summarizing the results of their positioning accuracy. It was reported that the positioning accuracy of flights based solely on onboard sensors was, at best, 8 cm. Ubellacker et al. [5] developed an aerial manipulation system with two cameras for object detection and localization, which is similar to our system, with a soft gripper equipped. The object localization error and flight positioning error at different flight speeds were determined to be at least 4 cm and 2 cm, respectively. However, it is impossible to avoid the unexpected collisions that occur between a finger and an object during grasping. Even though extensive studies have been conducted on aerial manipulation techniques [21,22,23], few studies delve deeply into camera-based object detection and localization in the context of aerial manipulation, as well as localization for UAV positioning. This highlights the need for further research on integrating these advanced sensors with control strategies aimed at ensuring precise and stable UAV operation during active manipulation.

In this study, we developed an aerial manipulation system that does not rely on an offboard tracking system to achieve self-contained autonomous aerial manipulation in a GPS-denied environment. The system integrates two types of onboard cameras and a multi-fingered robotic hand with proximity sensors attached directly the bottom of the UAV. The cameras are used to capture the target object to be grasped and for stable flight up to the proximal graspable area. The main contributions of this paper lie in the following:

- An experimental evaluation of an improved object detection and localization algorithm that is robust to the position and attitude uncertainties inherent to UAVs by integrating information from two cameras.
- Experimental verification of the feasibility of indoor aerial manipulation based on the integrated use of onboard cameras and proximity sensors.

Utilizing a robotic hand based on proximity sensors for aerial manipulation is an original technology pioneered by our research group. To our knowledge, the highly integrative use of cameras and proximity sensors, with different detection ranges, on UAV aerial manipulation is a unique attempt in this paper. This paper is the first to leverage the strengths of cameras and proximity sensors to propose a series of aerial manipulation scenarios and demonstrate its feasibility by the developed aerial manipulation system, including accurate object location detection, flight positioning, and grasping that can correct flight errors in the proximal region.

The remainder of the paper is organized as follows. Section 2 describes the aerial manipulation scheme by the UAV using cameras and proximity sensors. Section 3 presents the configuration of the aerial manipulation system used in this study. Section 4 explains the object detection and localization based on integrated two-camera information. Section 5 describes experiments to determine the on-board camera configuration for precise positioning flight. Section 6 shows the results and evaluation of the aerial manipulation experiment, including grasping based on robotic hand proximity sensor feedback. Section 7 presents the conclusions.

## 2. Aerial Manipulation Scenario

The UAV system developed in this paper featured proximity sensors attached to the robotic hand. This eliminates the problem of object occlusion by the hand or the target object going out of the field of view in close proximity, which is seen in most conventional systems that merely use cameras. This study assumes aerial manipulation in indoor environments under general lighting conditions.

**Figure 1:**
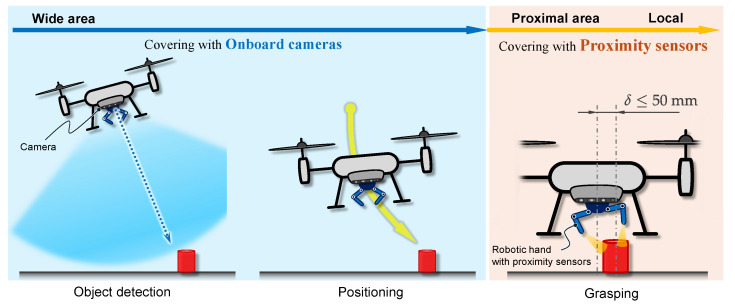
Aerial manipulation scheme adopted for the developed UAV system equipped with a multi-fingered robotic hand with proximity sensors.

Taking advantage of the different detection areas that cameras and proximity sensors have, we adopted the flow of aerial manipulation shown in Figure 1. A target object specified by the operator is extracted from the camera vision on the flying UAV, and its 3D position is obtained using cameras. Then, to approach the targeted object, the reference position of the UAV is set directly above the object at a certain height, and the UAV is navigated using the onboard camera. Once the robotic hand position converges to a graspable range around the object, proximity sensors inside the robotic hand are activated for the hand to pre-grasp while maintaining non-contact and transitioning to a grasping posture, providing stable grasping without knocking over or damaging the object. Finally, the object is transported to the desired place again using the camera.

**Figure 2:**
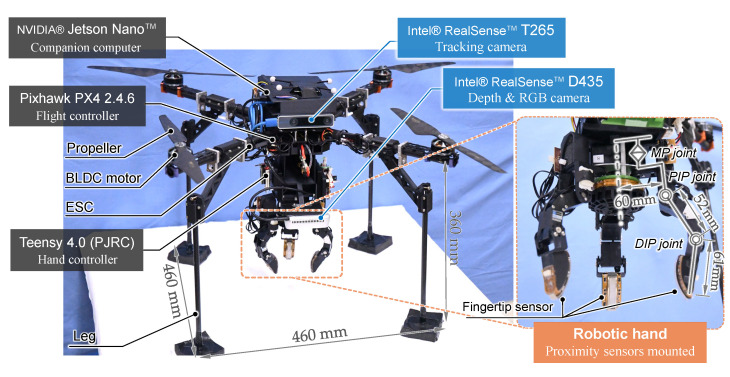
Overview of the developed UAV and multi-fingered robotic hand.

To determine the reliable grasping range of the robotic hand, we conducted a preliminary experiment using the same robotic hand used in this study, shown in Figure 2. The reliable grasping range is determined by the range of motion of the fingers and the detection range of the proximity sensors. The maximum opening width of the hand is 160 mm (80 mm on either side from the center of the hand). We verified the following ability of the fingertip proximity sensor-based pre-grasp-controlled single finger to the target plane, which was oscillated in the normal direction of the fingerpad, with an amplitude from 10 mm to 50 mm and a frequency from 0.1 Hz to 2.0 Hz. Due to the pre-grasp control, the fingertip follows the movement of the facing surface while maintaining a direct face and keeping a certain distance. The experimental results demonstrate that the finger could follow any oscillated plane while maintaining a distance without colliding with the surface when the target distance of the pre-grasp control was set to 30 mm. Based on the result, we determined the reliable grasping range to be 80 mm − 30 mm = 50 mm from the center axis of the robotic hand. Therefore, there are critical conditions for the UAV that must be met for the robotic hand to grasp an object successfully:

1. Positioning: The UAV must be positioned within a 50 mm radius around the object so that the robotic hand can grasp it reliably using proximity sensor information. To clarify, accurate flight positioning capability is required.
2. Stability: The UAV must maintain a stable hover to minimize the movement of the robotic hand while it is positioned around the object. In other words, high positioning precision is required.
3. Time allocation: The UAV must remain steady long enough for the robotic hand to complete the grasping action.

In this paper, we validated the UAV flight and aerial manipulation, specifically focusing on object detection and localization and flight positioning for approaching the object based on camera information.

## 3. System Configuration and Procedure for Aerial Manipulation

Figure 2 shows the overall configuration of our aerial manipulation system, which is equipped with two types of cameras and a multi-fingered robotic hand with proximity sensors. The total weight of the system is 3.46 kg, including the robot hand of 0.84 kg.

### 3.1. UAV Platform

The UAV structure is built based on a Tarot IRON MAN 650 platform with four axes; namely, the UAV is configured as a quadrotor. Each axis is equipped with a brushless DC motor and an electronic speed controller (ESC). The UAV is equipped with carbon fiber pipe legs for safety landing. Lithium polymer batteries for the electronic components, such as computers, cameras, and a robotic hand, were mounted on the platform. The UAV platform has space to install a battery for the motors; however, power for motors was supplied by an offboard power supply to ensure stable evaluation with experimental results that are not affected by voltage drops in the battery.

### 3.2. Multi-Fingered Robotic Hand Using Proximity Sensors

The right picture in Figure 2 shows the overview of the multi-fingered robotic hand for aerial manipulation developed based on our previous study [6]. Each finger is configured with two flexion joints and one pivot joint, resulting in a total of nine degrees of freedom in three fingers. A proximity sensor is attached to three fingertips. The sensor is a Net-Structured Proximity Sensor (NSPS) consisting of an analog network circuit with infrared photoreflector elements, which contains a light-emitting diode and a phototransistor in a package, arranged in an n×m grid. The amount of reflected infrared light received by each phototransistor varies depending on the distance and inclination between the finger and a nearby object, and the total current and current distribution of the NSPS change accordingly [24]. Using this principle, the multi-fingered hand can determine the appropriate grasping method without contacting the object.

The optical NSPS is marked by its high-speed response, allowing for it to obtain information on relative distance and orientation within 1 ms. Additionally, since there are no obstructions between the sensors inside the hand and the object, the occlusion problem that often troubles cameras can be solved. On the other hand, its detection range is relatively narrow. Thus, in a series of aerial manipulations, the UAV system needs to detect the target object from a wide area and flight close to it until the UAV goes within the detection range of the proximity sensors on the hand, relying on cameras.

### 3.3. Cameras

The onboard cameras to be used in our UAV system targeted in this study are required to have two functions: (1) the ability to detect the targeted object from a distance and localize its relative position, and (2) the ability for self-localization for navigation.

For object detection, we adopted the Intel^®^ RealSense™ D435 (Intel Corporation, Santa Clara, CA, USA) (hereafter called D435) camera [25], which boasts a wide measurement range. This sensor is equipped with an RGB camera and two depth sensors, obtaining real-time depth images using an active stereo method with an IR projector. The camera is capable of 3D position detection at a high frame rate, making it less likely to lose sight of the targeted object even when swaying due to external disturbances on a UAV.

As an alternative to a motion capture system fixed in the outside world, we adopted the Intel^®^ RealSense™ T265 (Intel Corporation, Santa Clara, CA, USA) (hereafter called T265) camera [26], which can be mounted on a UAV to enable self-localization. The T265 is equipped with a stereo fisheye camera, an IMU, and a built-in processor. The camera employs a VIO to provide an accurate real-time estimate of the camera’s position and motion without using markers. This fusion of data supports high-frequency updates, up to 200 Hz for the gyroscope, ensuring precise and responsive tracking in various environments.

### 3.4. System

**Figure 3:**
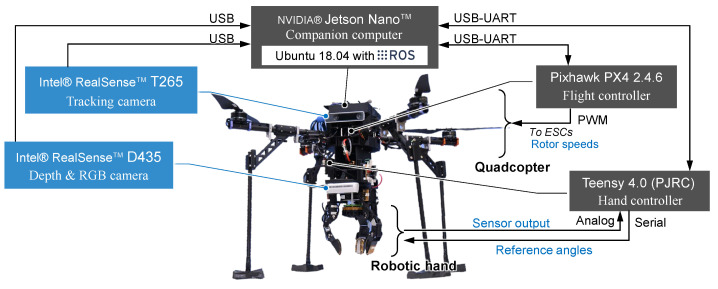
System architecture diagram of the aerial manipulation system.

The system architecture is illustrated in Figure 3. This study centers on UAV control and object detection, which involves advanced image processing. To handle these computationally intensive tasks, NVIDIA^®^ (Santa Clara, CA, USA) Jetson Nano is employed as a companion computer, providing the necessary processing power and efficiency for practical image analysis and real-time control. The two cameras are connected to the computer via USB. The companion computer has the Robot Operating System (ROS) installed, which performs the peripheral device management. The flight controller, Pixhawk PX4 2.4.6, is connected to the computer via UART communication to manage the UAV’s position and orientation control. Flight commands and current attitude are exchanged through the MAVROS package using the Micro Air Vehicle Link (MAVLink) communications protocol. The companion computer is operated via remote access from a ground station Windows computer.

The proximity sensor data acquisition and the angle control of the finger joint servo motors of the robotic hand are completed by a hand controller executed on a microcomputer without the need for a companion computer. The hand controller and companion computer are connected via UART communication, allowing for them to stream the output values of the proximity sensors.

### 3.5. Experimental Environment

All flight experiments were conducted in a 3 m cubic cage installed in a sufficiently large room. Note that the cage mesh is not detected by the two cameras and, therefore, does not affect the system’s positioning or object detection accuracy. The following constraints should directly impact the performance evaluation of the developed system intended for indoor aerial manipulation.

1. Environmental factors: External conditions, particularly including lighting, can affect camera accuracy. The camera used for object detection must be robust against light reflections to ensure precise measurements.
2. Ground effect: Most aerial manipulation involves picking up an object that has been placed on the ground, which can be exposed to ground effect and loss of stability while hovering over the object.

### 3.6. Procedure for Aerial Manipulation

**Figure 4:**
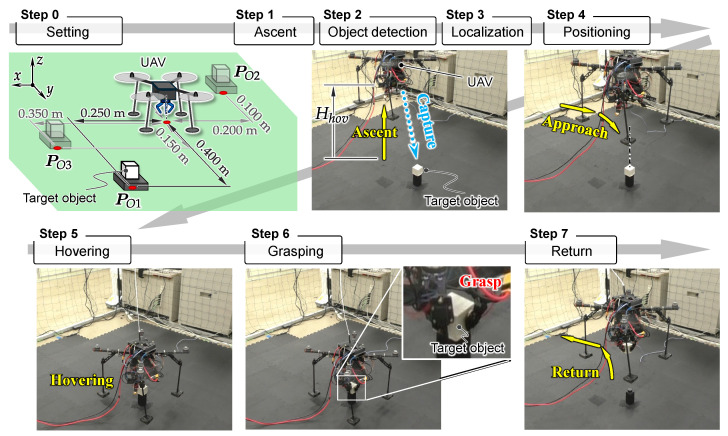
Aerial manipulation procedure.

The procedure for aerial manipulation is as follows, as shown in Figure 4.

1. Ascent: Ascend the UAV to a height of $H_{hov}$ from the ground.
2. Object detection: Identify the target object by using a D435 camera.
3. Localization: Determine the targeted object position relative to the UAV by using D435 and T265 during hovering only once.
4. Positioning: Navigate the UAV based on the self-localization using the T265 camera fused with the flight controller to align itself directly above the targeted object while maintaining a height of $H_{hov}$.
5. Hovering: Descend the UAV slowly until a height of $H_{grab}$ just above the object, then hover precisely over it for a time period $T_{hov}$.
6. Grasping: Grasp the object with the robotic hand using its proximity sensor technology while hovering.
7. Return: After grasping, return to its initial starting position while carrying the object.

In the UAV flight maneuvers, such as positioning and hovering, the UAV position and orientation are controlled using a PID controller within the flight controller to reach the target coordinates based on self-localization using the T265 camera.

## 4. Object Detection and Localization Using Onboard Cameras

**Figure 5:**
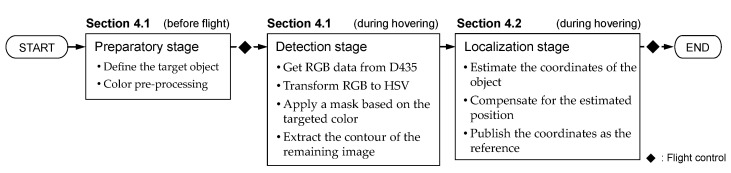
Schematic diagram of the object detection and localization algorithm.

To achieve autonomous aerial manipulation, we developed a solid object detection algorithm that accounts for the UAV’s movement imperfections to provide accurate coordinates for the target object. Figure 5 outlines the different steps involved in the object detection and localization algorithm using both the D435 and T265 cameras.

For object detection, we assume that the color of the object is known. The D435 camera mounted below the UAV body to face the ground was used for object detection, enabling it to detect and locate the target object. The OpenCV library was used for image processing on the companion computer.

### 4.1. Target Object Detection Using D435

The object detection method using the average HSV (Hue, Saturation, Value) value for image processing is effective in detecting a wide range of similar color objects. However, it may also detect objects of colors other than the specific color, leading to false positives.

It is essential to improve detection accuracy and success rate, as detecting the wrong object can pose significant dangers during aerial manipulations. To mitigate these issues, we introduced a pre-processing stage to the object detection algorithm. In this step, the user manually determines the HSV color of different objects (of different colors) and then stores these values in a file. This stage involves precisely determining the HSV value of the targeted object before the flight, ensuring that the detection is tailored specifically to the exact color of the object. Then, apply a mask based on the HSV value of the targeted object determined by the user at the beginning of the flight. Then, find the contours and draw a bounding box to the largest contour.

**Figure 6:**
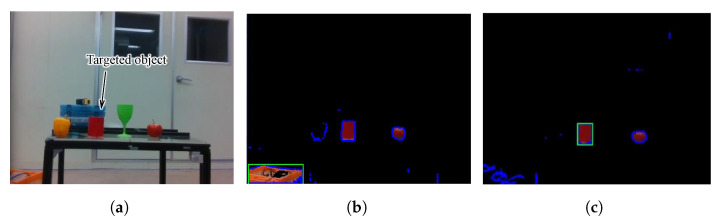
Object detection output including the color pre-processing stage: (**a**) the RGB output, (**b**) the RGB output after applying a mask without pre-processing step, (**c**) the RGB output after applying a mask with pre-processing step. The green boxes in (**b**,**c**) represent the bounding box and indicate detected objects by the algorithm.

Figure 6 presents the output of the algorithms with/without using the pre-processing step. In this experiment, the intended target was the red cylinder on the table. By utilizing the pre-processing step, the algorithm was able to detect the object of the intended color even in an environment with similarly colored objects, while one without the pre-processing step mistakenly identified the orange box instead due to poor accuracy. This process allows for more reliable and accurate detection of the intended object, even in complex environments with multiple-colored objects and varying lighting conditions.

### 4.2. Targeted Object Localization Using D435 and T265 Cameras

#### 4.2.1. Method

One of the typical classical object localization methods using RGB-D cameras is the intrinsic parameter-based method (hereafter referred to as the IP method). The IP method utilizes the camera’s intrinsic parameters, which are focal lengths $f_{x}$, $f_{y}$ and principal points $c_{x}$, $c_{y}$, to directly map 2D pixel coordinates to 3D real-world coordinates. When the depth camera is fixed, the targeted object coordinates (xOd,yOd,zOd) are obtained by normalizing the pixel coordinates $\left(\hat{x},\hat{y}\right)$ and scaling them by the detected depth $d_{O}$, representing the distance between the camera and object.

(1) xOd=x^dO=(u−cx)dOfx

(2) yOd=y^dO=(v−cy)dOfy

(3) zOd=dO

where (u,v) represents the horizontal and vertical pixel coordinate in the image.

**Figure 7:**
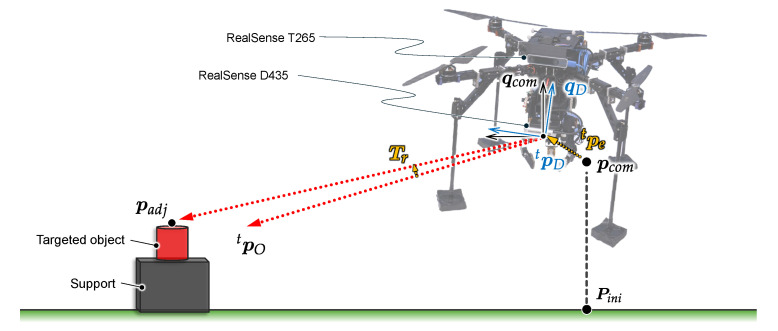
Compensation for object localization based on UAV position and orientation (represented on a 2D plane for clarity).

To improve the accuracy of object localization, addressing the inherent instability of tilting and drifting caused by external factors like the ground effect or internal factors like motor vibrations, we leveraged real-time attitude data from the T265 camera on the UAV’s movements. Figure 7 illustrates the object localization compensation method. Here, the relative coordinates of the object with respect to the UAV are obtained, and the command is given to be incremented to the initial hovering position.

The orientation of the UAV is defined using a quaternion expressed as Equation (4).

(4) $$q_{D}=q_{x}i+q_{y}j+q_{z}k+q_{w}$$

where $q_{x}$, $q_{y}$, and $q_{z}$ are the components of the imaginary vector, $q_{w}$ is the scalar component, and i, j, and k are the imaginary units. The relative quaternion $q_{rel}:=\left(q_{xr},q_{yr},q_{zr},q_{wr}\right)$, which represents the rotation difference between the commanded quaternion $q_{com}$ and the UAV’s actual orientation, is expressed as Equation (5).

(5) $$q_{rel}=q_{com}^{-1}⊗q_{D}$$

where the operation symbol ⊗ represents the Kronecker product. Using $q_{rel}$, we can determine the rotation matrix associated with the current orientation.

(6) $$\begin{array}{c} T_{r}=\left(\begin{array}{ccc} 1-2(q_{yr}^{2}+q_{zr}^{2}) & 2(q_{xr}q_{yr}-q_{zr}q_{wr}) & 2(q_{xr}q_{zr}+q_{yr}q_{wr})\\ 2(q_{xr}q_{yr}+q_{zr}q_{wr}) & 1-2(q_{xr}^{2}+q_{zr}^{2}) & 2(q_{yr}q_{zr}-q_{xr}q_{wr})\\ 2(q_{xr}q_{zr}-q_{yr}q_{wr}) & 2(q_{yr}q_{zr}-q_{xr}q_{wr}) & 1-2(q_{xr}^{2}+q_{yr}^{2}) \end{array}\right) \end{array}$$

To convert the object coordinates from the D435 camera system to the T265 camera system, a transformation matrix $T_{t}$ is introduced and expressed in Equation (7).

(7) pOt=TtpOd

where,

(8) $$\begin{array}{c} T_{t}=\left(\begin{array}{ccc} 1 & 0 & 0\\ 0 & 0 & 1\\ 0 & -1 & 0 \end{array}\right), \end{array}$$

pOd=(xOd,yOd,zOd)T represents the coordinates of the targeted object determined using the D435 image, and pOt=(xOt,yOt,zOt)T represents it in the T265 coordinate system.

By applying the rotation matrix $T_{r}$ and introducing the translational position error pet=pDt−pcomt that takes into account the positional error of the UAV regarding of the command, we effectively adjust the object’s coordinates by Equation (9).

(9) padj=TrpOt+pet

#### 4.2.2. Experimental Evaluation

To verify object localization using D435 and T265 cameras, we conducted a object detection experiment using a hovering UAV. A cylindrical red object was used as a target object. The UAV hovered with the flight command coordinates of pcomt=(xcomt,ycomt,zcomt)T$={\left(0,\;0,\;0.35\right)}^{T}$ [m] while the targeted object placed some distance away from the UAV; in this experiment, $P_{O}=\left(x_{O},y_{O}\right)=\left(-0.107,\;0.270\right)$ [m] from the initial position. The target object was identified using the D435 at the hovered position, and its relative position coordinates with the UAV were calculated using the proposed algorithm. To evaluate the object localization accuracy, multiple computations were executed during 10 s of hovering.

**Figure 8:**
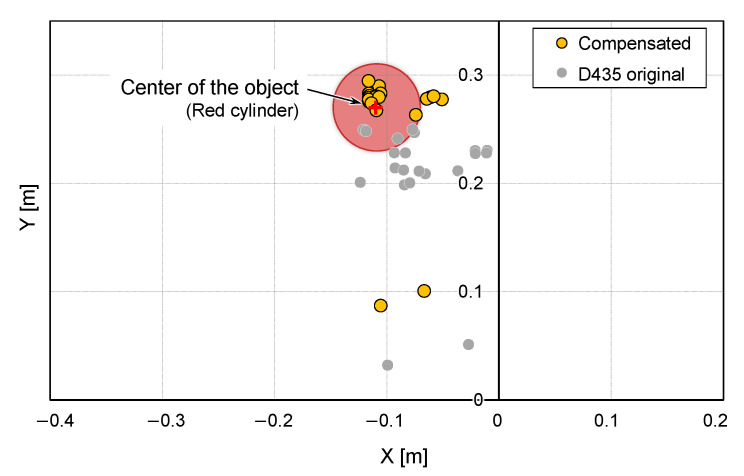
Targeted object position estimation before and after compensation.

The object localization results are shown in Figure 8. During hovering, the T265 camera returned a positional error pet of up to 37 mm in the x-coordinate and up to −9 mm in the y-coordinate. The raw output from the D435 camera was scattered and inaccurate, largely due to the sensitivity of the camera to the UAV’s attitude variations during the flight. By applying the proposed method, the detected coordinates are successfully compensated to align closely with the actual position. As a result, although the algorithm doesn’t solve the presence of outliers, which can be caused by a poor detection of the distance to the object due to the imperfect calibration of the camera or reflection problems, we achieved localization with sufficient accuracy for our usage even when the UAV was not perfectly stable.

## 5. UAV Positioning Using Onboard T265 Camera

The T265 camera has been employed in various research scenarios, such for the localization of terrestrial mobile robots [27,28] and state estimations for aerial [29,30] and underwater vehicles [31]. Visual SLAM using this product is performed based on image features in the video frames [32]. Therefore, the mounting orientation of the camera on the robot base should affect the accuracy of self-localization; however, its orientation, i.e., configuration—down-facing or front-facing—has not been directly compared in the context of aerial manipulation. Understanding which configuration yields more precise and stable flight control is crucial for enhancing our system.

### 5.1. Determination of Camera Orientation

The features of each configuration of the T265 camera in terms of its use for aerial manipulation can be described as follows.

• Down-facing configuration:

·

Enhance the UAV’s ability to locate and maintain its position over a target object, especially in applications requiring precise hovering above the object.

·

Allows for work to be performed integrally in tandem with the D435 for SLAM, providing detailed environmental mapping and precise localization by leveraging both cameras’ data streams [33].

·

Potentially difficult to take sufficient features from the floor to estimate its position.

·

Occlusion issues can be caused by including a robotic hand in the field of view.
• Front-facing configuration:

·

Effectively estimate its position due to sufficient changes in scenery and environmental features.

·

Less likely that the field of vision will be obstructed by the UAV’s body or components.

·

Hard to capture the targeted object in the field of view when the UAV approaches above it.

Given these considerations, we explored which configurations provide better positional accuracy and flight stability for our UAV during aerial manipulation tasks.

**Figure 9:**
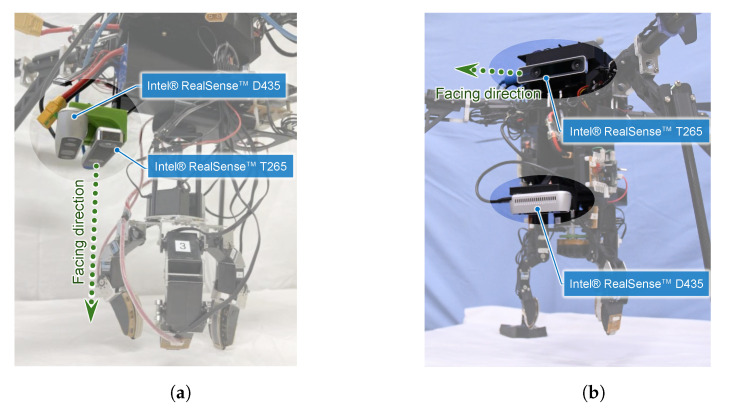
Camera configurations: (**a**) down-facing configuration, (**b**) front-facing configuration.

We conducted a series of flight experiments involving object approach and homing, except for the grasping step, using the UAV with different T265 camera orientations, down-facing and front-facing, as shown in Figure 9. The ascent height was set to $H_{hov}=0.7$ m, the grasping altitude was set to $H_{grab}=0.02$ m, and the hovering period was set to $T_{hov}=15$ s. The attitude sampling rate of the T265 camera was set to the default setting of 30 Hz.

To interpret the experimental results, we employed two metrics, the Mean Distance Error (MDE) and the Twice the Distance Root Mean Square (2DRMS). The MDE represents the distance between the reference coordinates and the mean of the actual position coordinates, and a two-dimensional (2D) MDE is defined by Equation (10):

(10) $$\delta =\sqrt{\delta_{x}^{2}+\delta_{y}^{2}}$$

where,

(11) $$\delta_{a}=\frac{1}{N}\sum_{i=1}^{N}\left(a_{i}-a_{ref,i}\right)$$

is the mean error of the *a* coordinate (*a* is replaced by *x* or *y*), *N* is the sample size, and $a_{i}$ and $a_{ref,i}$ are the observed and reference locations, respectively, for the *i*th sample (i=1,2,…,N). MDE can be used as a metric of UAV positioning accuracy, and a smaller MDE indicates a higher UAV positioning accuracy.

The 2D DRMS is a measure of the mean error magnitude in position estimation, as calculated using Equation (12).

(12) $$DRMS=\sqrt{\sigma_{x}^{2}+\sigma_{y}^{2}}$$

where $\sigma_{a}$ is the standard deviation of the *a*-axis (*a* is replaced by *x* or *y*) displacement calculated using Equation (13).

(13) $$\sigma_{a}=\sqrt{\frac{1}{N}\sum_{i=1}^{N}{\left(a_{i}-\bar{a}\right)}^{2}}$$

where $\bar{a}$ is the sample mean. 2DRMS=2×DRMS represents the radius within which approximately 95% of all positional errors fall, whereas DRMS represents the radius that contains 68% of the probability. DRMS can indicate the UAV positioning flight precision. These statistical measures are commonly used in fields such as ballistics and GPS-based navigation to quantify positional accuracy and error [34], and they make it a valuable metric for assessing the reliability and precision of positioning systems.

**Figure 10:**
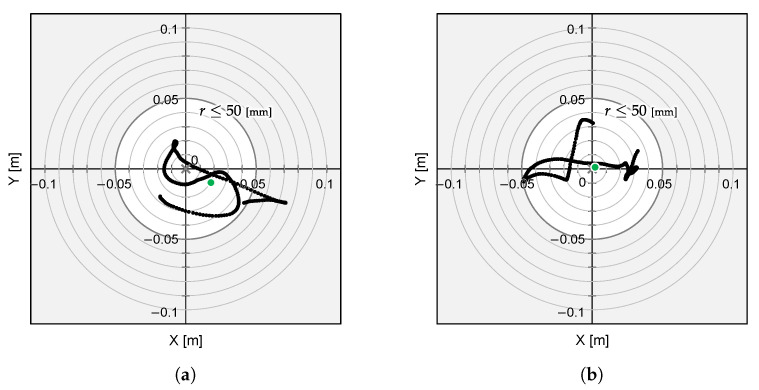
Flight experiment result using (**a**) the down-facing configuration and (**b**) the front-facing configuration. The grey cross markers indicate the object’s position and the green dots represent the mean position of the UAV’s flight path.

As a result of the experiments, both camera configuration demonstrations were conducted successfully. The position coordinates of the flying UAV are depicted in Figure 10. The 2D planar MDE, δ, and 2DRMS are summarized in Table 1, which were evaluated during a 7 s hovering directly over the object that was localized using cameras.

The results indicate that the positioning errors by the front-facing configuration are significantly reduced, and suggest that this configuration provides a more accurate positioning solution for our UAV system. While the visual odometry relying on floor-based features alone in a down-facing configuration leads to a lack of accuracy of the UAV’s positioning, the front-facing configuration allows for more accurate localization due to the increased visibility and availability of features in the environment. By offering lower positional errors, the front-facing camera orientation ensures better flight accuracy during aerial manipulation tasks. However, even the front-facing configuration has a 2DRMS error, superior to 50 mm, which means that the flight stability is insufficient and the robotic hand requirement is not fulfilled yet.

### 5.2. Determination of the Attitude Sampling Rate

To improve the 2DRMS further, we conducted multiple flight experiments to determine the attitude sampling rate of the T265 camera, which potentially affects flight precision. The T265 camera was oriented towards a front-facing and attitude sampling rate set to 30, 80, 150 and 200 Hz. The experiments followed the same procedures described above.

The results of the 2DRMS measured during the UAV’s hovering phase over the targeted object are summarized in Table 2. The results indicate that increasing the attitude sampling rate leads to a notable reduction in 2DRMS. A higher sampling rate enables more rapid and precise adjustments of the UAV’s estimated position, which enhances the overall stability and accuracy of the system. As a result, the system can better account for dynamic changes and correct deviations in real time, leading to a significant reduction in the 2DRMS. Finally, we obtained a more accurate and stable system, with a 2DRMS of 27.1 mm when the attitude sampling rate was 200 Hz, which meets the requirement given in Section 2.

Based on these results, we determined to orient the T265 camera into a front-facing position and set the attitude sampling rate to 200 Hz.

## 6. Experiments on Aerial Manipulation

Through the flight experiments, we verified the feasibility of aerial manipulation using our UAV system. More precisely, we evaluated whether the developed UAV system, including the object detection algorithm and positioning flight based on onboard cameras, is sufficient to achieve our main goal: reliable and precise aerial manipulation.

### 6.1. Experimental Method

As a target grasping object in experiments, a white cube with side lengths of 60 mm and a mass of 0.15 kg, which ensured reliable detection and grasping by the hand’s proximity sensors, was employed. The ascent height was set to $H_{hov}=0.45$ m, and the hovering period was set to $T_{hov}=15$ s.

To evaluate the precision of the UAV positioning and the object detection algorithm more effectively, we prepared three different object placement positions, $P_{O1}=\left(x_{O1},y_{O1}\right)=\left(0.250,\;0.400\right)$ [m], $P_{O2}=\left(x_{O2},y_{O2}\right)=\left(-0.200,\;-0.100\right)$ [m], and $P_{O3}=\left(x_{O3},y_{O3}\right)=\left(0.350,\;0.150\right)$ [m], relative to the UAV initial position, as illustrated in the **Step 0** in Figure 4. We conducted six aerial manipulation experiments for three different object placement positions.

### 6.2. Results

**Figure 11:**
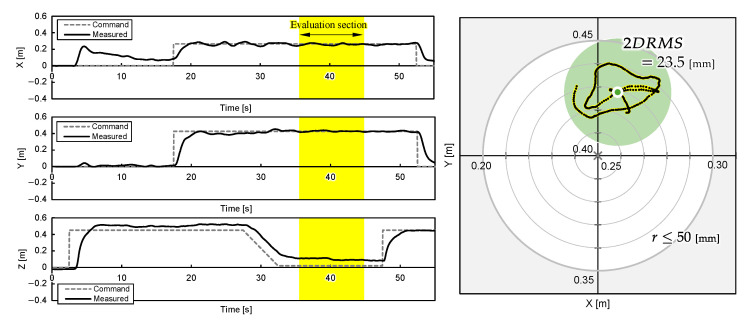
UAV attitudes during aerial manipulation Experiment 1: object at $P_{O1}=\left(x_{O1},y_{O1}\right)=\left(0.250,0.400\right)$ [m].

**Figure 12:**
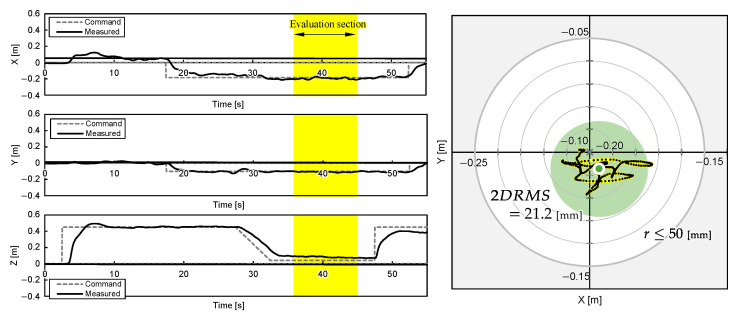
UAV attitudes during aerial manipulation Experiment 2: object at $P_{O2}=\left(x_{O2},y_{O2}\right)=\left(-0.200,-0.100\right)$ [m].

**Figure 13:**
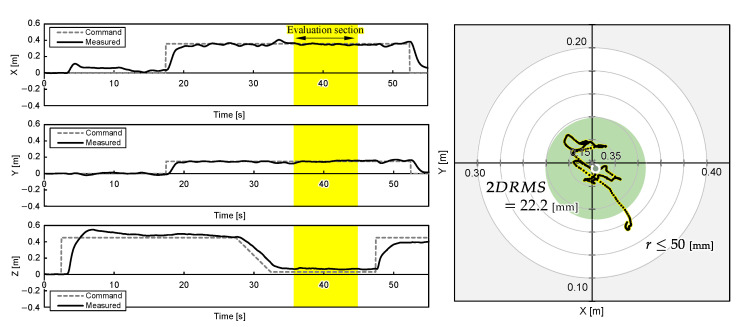
UAV attitudes during aerial manipulation Experiment 3: object at $P_{O3}=\left(x_{O3},y_{O3}\right)=\left(0.350,0.150\right)$ [m].

One experimental result for each object position is shown in Figure 11, Figure 12 and Figure 13. The grey cross markers in the right graphs indicate the object’s position and the green dots represent the mean position of the UAV’s flight path. The green-shaded region in the right graphs illustrates the region of 2DRMS. The snapshots in Figure 4 show a series of aerial manipulations performed in this experiment. In all experiments in different object placements, the UAV aerial manipulation system was able to grasp the objects, confirming the feasibility of aerial manipulation with the developed system. From these results, we can observe that the UAV hovered accurately around the position of the object, as determined by the D435 and T265 onboard cameras, and the UAV’s hovering appears relatively stable.

The 2D planar MDE, δ, and 2DRMS during 10 s hovering, which are shaded yellow in Figure 11, Figure 12 and Figure 13, and the time the flight height stabilized 3.5 s after the hovering command was sent, are evaluated and summarized in Table 3. The results indicate that the average MDEs for Experiment 2 and 3 was sufficiently small compared to the graspable range of the robotic hand, and the positioning accuracy achieved 22.9 mm. The evaluated DRMS and 2DRMS indicated a 68% probability that the UAV stays within a radius of a 11.2 mm circle and a 95% probability that it stays within a radius of a 22.4 mm circle. Figure 11, Figure 12 and Figure 13 show that, during grasping stage in the three trials, the center of the robotic hands was within a 50 mm radius, which is the graspable range of the robotic hand. Overall, the results suggest that our UAV system can realize aerial manipulation by staying within the graspable range of the robotic hand during the entire grasping period; therefore, the requirement was fulfilled by using the proposed methods. However, the MDE in Experiment 1 is relatively large, as shown in Table 3. One possible cause of this issue was the drift in the built-in IMU during localization by T265, resulting in inaccuracies in self-location. This positional error value is almost consistent with the results of related studies [5] using similar systems, suggesting the need for further improvement of the VIO method. On the other hand, the experimental results also reveal that the positional error of the developed aerial manipulation system outperforms that of other existing studies using visual sensors [14,20].

## 7. Conclusions

In this study, we developed a UAV system for autonomous aerial manipulation using onboard cameras for object localization and UAV positioning, as well as a multi-fingered robotic hand with proximity sensors for stable grasping. We developed an object detection algorithm that compensates for onboard camera information robust to inherent instability caused by external disturbances, thereby improving the accuracy of object localization despite the imperfect movement of the UAV. We also improved flight accuracy by determining the facing orientation of the camera used for the visual odometry and the attitude sampling rate. The aerial manipulation experiment using the developed UAV system demonstrates that the system could maintain a 95% probability for staying within a radius of 50 mm, which is the robotic hand’s requirement.

As a future challenge, we aim to apply the system for more complex aerial manipulation tasks, including grasping a moving object.

## Figures and Tables

**Table 1:** Down-facing and front-facing approach performance comparison.

	Down-Facing	Front-Facing
MDE δ [mm]	20.4	2.4
2DRMS [mm]	60.6	59.0

**Table 2:** Evolution of the 2DRMS with the T265 attitude sampling rate.

Sampling rate [Hz]	30	80	150	200
2DRMS [mm]	59.0	44.5	30.4	27.1

**Table 3:** Aerial manipulation performance evaluation.

		Experiment 1 $P_{O1}$	Experiment 2 $P_{O2}$	Experiment 3 $P_{O3}$	Average
MDE δ [mm]	Ave.	44.0	11.0	13.7	22.9
	Max.	57.5	17.4	17.9	
	Min.	28.9	2.9	2.9	
DRMS [mm]	Ave.	11.8	11.2	10.4	11.2
	Max.	19.1	15.0	22.6	
	Min.	5.7	8.0	3.6	
2DRMS [mm]	Ave.	23.7	22.4	20.9	22.4
	Max.	38.2	30.1	45.2	
	Min.	11.5	16.1	7.3	

## Data Availability

The original contributions presented in the study are included in the article; further inquiries can be directed to the corresponding author.
